# Evaluating Postoperative Morbidity and Outcomes of Robotic-Assisted Esophagectomy in Esophageal Cancer Treatment—A Comprehensive Review on Behalf of TROGSS (The Robotic Global Surgical Society) and EFISDS (European Federation International Society for Digestive Surgery) Joint Working Group

**DOI:** 10.3390/curroncol32020072

**Published:** 2025-01-28

**Authors:** Yogesh Vashist, Aman Goyal, Preethi Shetty, Sergii Girnyi, Tomasz Cwalinski, Jaroslaw Skokowski, Silvia Malerba, Francesco Paolo Prete, Piotr Mocarski, Magdalena Kamila Kania, Maciej Świerblewski, Marek Strzemski, Luis Osvaldo Suárez-Carreón, Johnn Henry Herrera Kok, Natale Calomino, Vikas Jain, Karol Polom, Witold Kycler, Valentin Calu, Pasquale Talento, Antonio Brillantino, Francesco Antonio Ciarleglio, Luigi Brusciano, Nicola Cillara, Ruslan Duka, Beniamino Pascotto, Juan Santiago Azagra, Mario Testini, Adel Abou-Mrad, Luigi Marano, Rodolfo J. Oviedo

**Affiliations:** 1 Department of Surgery, Organ Transplant Center for Excellence, Center for Liver Diseases and Oncology, King Faisal Specialist Hospital and Research Center, Riyadh 12271, Saudi Arabia; y.vashist@kfu.edu.sa; 2 Department of General Surgery, Mahatma Gandhi Medical College and Research Institute, Pondicherry, Cuddalore Rd, ECR, Pillayarkuppam, Puducherry 607402, India; doc.aman.goyal@gmail.com; 3 Department of Surgery, Adesh Institute of Medical Sciences and Research, Bathinda 151001, India; 4 Kasturba Medical College, Manipal, Manipal Academy of Higher Education, Manipal 576104, India; preethi.sshetty@manipal.edu; 5 Department of General Surgery and Surgical Oncology, “Saint Wojciech” Hospital, “Nicolaus Copernicus” Health Center, 80-000 Gdańsk, Poland; sgirnyi@copernicus.gda.pl (S.G.); tcwalinski@copernicus.gda.pl (T.C.); j.skokowski@amisns.edu.pl (J.S.); s.malerba10@studenti.uniba.it (S.M.); pmocarski@copernicus.gda.pl (P.M.); mkania@copernicus.gda.pl (M.K.K.); mswierblewski@copernicus.gda.pl (M.Ś.); 6 Department of Medicine, Academy of Applied Medical and Social Sciences-AMiSNS: Akademia Medycznych I Spolecznych Nauk Stosowanych, 52-300 Elbląg, Poland; k.polom@amisns.edu.pl; 7 Department of Precision and Regenerative Medicine and Ionian Area, University of Bari “Aldo Moro”, 70110 Bari, Italy; francesco.prete@uniba.it (F.P.P.); mario.testini@uniba.it (M.T.); 8 Department of Anesthesiology and Intensive Care, “Saint Wojciech” Hospital, “Nicolaus Copernicus” Health Center, 80-000 Gdańsk, Poland; mstrzemski@copernicus.gda.pl; 9 Department of Bariatric Surgery, UMAE Hospital de Especialidades del Centro Medico Nacional de Occidente, Guadalajara 44100, Mexico; osvaldo.suarez@academico.udg.mx; 10 Department of Surgery, Universidad de Guadalajara, Guadalajara 44340, Mexico; 11 Department of Surgery, Complejo Asistencial Universitario de Palencia, 34401 Palencia, Spain; jherrerak@saludcastillayleon.es; 12 Department of Medicine, Surgery, and Neurosciences, University of Siena, 53100 Siena, Italy; natale.calomino@unisi.it; 13 Department of Surgical Oncology & Robotic services, HCG Manavata Cancer Center, Nashik 422002, India; drvikas@manavatacancercentre.com; 14 Department of Gastrointestinal Surgical Oncology, Greater Poland Cancer Centre, 61-866 Poznan, Poland; witold.kycler@wco.pl; 15 Department of Surgery, University of Medicine and Pharmacy Carol Davila, 010001 Bucharest, Romania; valentin.calu@umfcd.ro; 16 Department of Surgery, Pelvic Floor Center, AUSL-IRCCS Reggio Emilia, 42122 Reggio Emilia, Italy; pasquale.Talento@ausl.re.it; 17 Department of Surgery, Antonio Cardarelli Hospital, 80131 Naples, Italy; antonio.brillantino@aocardarelli.it; 18 Department of General Surgery and Hepato-Pancreato-Biliary (HPB) Unit-APSS, 38121 Trento, Italy; francesco.ciarleglio@apss.tn.it; 19 Division of General, Oncological, Mini-Invasive and Obesity Surgery, University of Study of Campania “Luigi Vanvitelli”, 80131 Naples, Italy; luigi.brusciano@unicampania.it; 20 Department of Surgery, “SS. Trinità” Hospital, 09121 Cagliari, Italy; nicola.cillara@aslcagliari.it; 21 Department of Surgery, Dnipro State Medical University, 49044 Dnipro, Ukraine; 408@dmu.edu.ua; 22 Department of General and Minimally Invasive Surgery (Laparoscopy & Robotic), Centre Hospitalier de Luxembourg, 1210 Luxembourg, Luxembourg; pascotto.b@ch.lu (B.P.); azagra.JS@chl.lu (J.S.A.); 23 Department of Surgery, Centre Hospitalier Universitaire d’Orléans, 45000 Orléans, France; adel.abou-mrad@orange.fr; 24 Department of Surgery, Nacogdoches Medical Center, Nacogdoches, TX 75962, USA; 25 Department of Surgery, University of Houston Tilman J. Fertitta Family College of Medicine, Houston, TX 77001, USA; 26 Department of Surgery, Sam Houston State University College of Osteopathic Medicine, Conroe, TX 77301, USA

**Keywords:** robotic-assisted esophagectomy, esophageal cancer, minimally invasive surgery, fluorescence-guided technologies, postoperative outcomes

## Abstract

Background: Esophageal cancer, the seventh most common malignancy globally, requires esophagectomy for curative treatment. However, esophagectomy is associated with high postoperative morbidity and mortality, highlighting the need for minimally invasive approaches. Robotic-assisted surgery has emerged as a promising alternative to traditional open and minimally invasive esophagectomy (MIE), offering potential benefits in improving clinical and oncological outcomes. This review aims to assess the postoperative morbidity and outcomes of robotic surgery. Methods: A comprehensive review of the current literature was conducted, focusing on studies evaluating the role of robotic-assisted surgery in esophagectomy. Data were synthesized on the clinical outcomes, including postoperative complications, survival rates, and recovery time, as well as technological advancements in robotic surgery platforms. Studies comparing robotic-assisted esophagectomy with traditional approaches were analyzed to determine the potential advantages of robotic systems in improving surgical precision and patient outcomes. Results: Robotic-assisted esophagectomy (RAMIE) has shown significant improvements in clinical outcomes compared to open surgery and MIE, including reduced postoperative pain, less blood loss, and faster recovery. RAMIE offers enhanced thoracic access, with fewer complications than thoracotomy. The RACE technique has improved patient recovery and reduced morbidity. Fluorescence-guided technologies, including near-infrared fluorescence (NIRF), have proven valuable for sentinel node biopsy, lymphatic mapping, and angiography, helping identify critical structures and minimizing complications like anastomotic leakage and chylothorax. Despite these benefits, challenges such as the high cost of robotic systems and limited long-term data hinder broader adoption. Hybrid approaches, combining robotic and open techniques, remain common in clinical practice. Conclusions: Robotic-assisted esophagectomy offers promising advantages, including enhanced precision, reduced complications, and faster recovery, but challenges related to cost, accessibility, and evidence gaps must be addressed. The hybrid approach remains a valuable option in select clinical scenarios. Continued research, including large-scale randomized controlled trials, is necessary to further establish the role of robotic surgery as the standard treatment for resectable esophageal cancer.

## 1. Introduction

Esophagectomy remains a cornerstone in the curative treatment of esophageal cancer, the seventh most prevalent malignancy globally, despite significant advancements in multimodal approaches aimed at enhancing patient survival and quality of life [1]. For instance, incorporating neoadjuvant chemotherapy or chemoradiotherapy has demonstrated improved outcomes, with 5-year survival rates reaching up to 50% compared to surgery alone [2]. However, esophagectomy is a technically demanding procedure associated with substantial postoperative morbidity and mortality, which can negatively impact recovery, survival, and quality of life [3].

To mitigate surgical trauma, minimally invasive esophagectomy (MIE), employing thoracoscopic or laparoscopic techniques, has gained widespread acceptance, now constituting over two-thirds of esophageal surgeries [4]. These approaches have emerged as viable alternatives to traditional open surgery, offering significant benefits such as reduced postoperative pain, a lower incidence of pneumonia, and faster recovery times, all without compromising overall survival (OS) or disease-free survival (DFS) [5,6,7,8,9,10,11]. Nonetheless, open esophagectomy continues to be preferred in certain clinical scenarios due to its shorter operative times while maintaining comparable oncologic outcomes [12].

The advent of robotic-assisted surgery has further revolutionized minimally invasive techniques. The da Vinci Surgical System, approved by the U.S. Food and Drug Administration in 2000, marked a breakthrough in surgical innovation [13]. In 2004, Kernstine and colleagues performed the first robotic-assisted esophagectomy, demonstrating its feasibility [14]. Since then, the field of robotic surgery has expanded substantially with the introduction of newer platforms such as the Hugo™ system by Medtronic and the Versius system by CMR Surgical [15]. These advancements have broadened the scope of minimally invasive surgery, providing surgeons with additional tools to optimize outcomes in complex esophageal resections.

Despite the growing global adoption of robotic-assisted esophagectomy, it has yet to achieve widespread acceptance as a standard treatment modality for resectable esophageal cancer. Significant barriers to broader implementation include the high costs associated with acquiring and maintaining robotic systems, as well as a paucity of robust evidence unequivocally demonstrating its superiority over conventional surgical techniques [16]. Consequently, many centers have integrated hybrid surgical approaches that combine minimally invasive and open techniques. A prevalent practice involves performing the abdominal phase laparoscopically while utilizing open thoracotomy for the thoracic phase, thereby leveraging the advantages of minimally invasive surgery, such as reduced postoperative pain and shorter hospitalization, while ensuring oncologically effective resections [17].

This review aims to comprehensively assess the clinical and oncological outcomes of robotic-assisted esophagectomy. It seeks to elucidate emerging surgical trends, critically evaluate the advantages and limitations of robotic platforms, and analyze the learning curve associated with adopting this technology in the treatment of resectable esophageal cancer.

## 2. Materials and Methods

We conducted a comprehensive literature search using multiple online databases, including PubMed, Embase, Web of Science, and the Cochrane Library, to identify studies on robotic-assisted esophagectomy (RAE) published up to June 2024. The search strategy utilized a combination of subject headings and text words to ensure broad yet focused retrieval of relevant articles. Keywords and medical subject heading (MeSH) terms included but were not limited to “robotic”, “esophagectomy”, “minimally invasive surgery”, “thoracoscopy”, “laparoscopy”, and “esophageal cancer”. In addition, the references of eligible articles were manually reviewed to identify supplementary studies that met the inclusion criteria.

We included studies focusing on patients undergoing robotic-assisted esophagectomy for esophageal cancer, evaluating robotic-assisted techniques either as standalone procedures or as part of hybrid approaches. Comparative studies examining conventional approaches, such as minimally invasive esophagectomy (MIE) and open esophagectomy (OE), were also included. Eligible studies reported clinical outcomes such as overall survival and disease-free survival, perioperative metrics like operative time and blood loss, postoperative recovery, and complication rates. Retrospective and prospective studies, as well as case series and cohort studies involving ≥10 patients, were considered, provided they were published in English. Studies were excluded if they were reviews, systematic reviews, meta-analyses, or study protocols; case reports with fewer than 10 patients; studies lacking full-text availability; or non-human studies or those not reporting surgical or oncological outcomes. This focused yet inclusive methodology ensured a comprehensive analysis of the existing evidence base while maintaining relevance to the review objectives.

## 3. Postoperative Outcomes of Robotic Esophagectomy

Esophagectomy is widely recognized as one of the most challenging cancer surgeries, alongside pancreatectomy and hepatectomy. Despite advancements in perioperative care, surgical techniques, and anesthetic protocols that have contributed to reduced complication rates, esophagectomy continues to be a formidable procedure [18]. The average five-year survival rate for operable esophageal cancer remains approximately 28%, underscoring the significant impact of surgical complications on patients’ quality of life, particularly in the context of a limited life expectancy [18,19].

To standardize the reporting and stratification of postoperative complications, several international organizations, including the Esophagectomy Complications Consensus Group (ECCG), the Dutch Upper Gastrointestinal Cancer Audit (DUCA), and the Oesophago-Gastric Anastomosis Audit (OGAA), have established outcome measures [11,20,21]. The Clavien–Dindo (CD) classification system remains a universally accepted tool among these groups for categorizing postoperative complications [22].

The Traditional Invasive vs. Minimally Invasive Esophagectomy (TIME) trial in 2012 marked a pivotal shift toward the adoption of minimally invasive techniques in esophagectomy [5]. Subsequent prospective studies, such as those conducted by the DUCA group in 2017, demonstrated improved “textbook outcomes” for minimally invasive esophagectomy compared to the open approach (odds ratio: 1.60 [1.31–1.94], *p* = 0.004) [21]. Robotic-assisted minimally invasive esophagectomy (RAMIE) has since gained traction due to its promising initial results. However, debates persist regarding its superiority over conventional minimally invasive esophagectomy (cMIE).

Two key randomized controlled trials (RCTs) have sought to address this question. The Dutch ROBOT trial aimed to detect a 22% absolute risk reduction in CD grade ≥ 2 complications with RAMIE compared to open transthoracic esophagectomy (OTE). RAMIE demonstrated a lower rate of surgery-related postoperative complications (CD grade ≥2) compared to OTE (59% vs. 80%; RR 0.74, 95% CI: 0.57–0.96, *p* = 0.02) [1]. In contrast, the multicenter RAMIE trial conducted by a Chinese group compared RAMIE to cMIE for esophageal squamous cell carcinoma. Their findings revealed no significant difference in CD grade ≥ 2 complications between RAMIE (12.2%) and cMIE (10.2%) (RR 1.20, 95% CI: 0.66–2.15, *p* = 0.551) [23].

Meta-analyses have provided additional insights into postoperative outcomes. Esagian et al. (2022) included five retrospective studies, four prospective studies, and one RCT comparing RAMIE with OTE. The analysis found no statistically significant difference in overall complication rates between the RAMIE group (27.88%) and the OTE group (33.93%) (OR: 0.66, 95% CI: 0.42–1.05, *p* = 0.08) [24]. Perry et al. further expanded this analysis to include 18,187 patients, offering a broader perspective on short-term outcomes between RAMIE and cMIE [25].

Specific surgical approaches have also been analyzed. Zhou et al. (2022) focused on McKeown esophagectomy, pooling data from seven propensity-matched retrospective studies. Their findings indicated no significant difference in overall complications between RAMIE and cMIE (OR: 1.10, 95% CI: 0.86–1.41, *p* = 0.46) [26]. Meanwhile, Angeramo et al. conducted a meta-analysis of 60 studies examining Ivor Lewis esophagectomy, reporting significantly lower overall morbidity rates with RAMIE (30%, 95% CI: 24–38%) compared to cMIE (40%, 95% CI: 34–47%; OR: 0.67, 95% CI: 0.58–0.79, *p* < 0.001) [27].

Real-world data from the OGAA group have highlighted postoperative outcomes on a global scale, encompassing over 137 countries. Their analysis emphasized the critical role of anastomotic leaks and conduit necrosis in influencing patient mortality, providing a comprehensive overview of postoperative risks and outcomes [11].

### 3.1. Clinical Recovery Metrics

#### 3.1.1. Length of Hospital Stay

The length of hospital stay (LOS) is a critical postoperative outcome indicator, often reflecting the overall recovery and complication rates. In the ROBOT trial, the median ICU stay was reported as one day in both the RAMIE and OTE groups (*p* = 0.45). The median hospital stay was slightly shorter in the RAMIE group (14 days) compared to the OTE group (16 days), but this difference was not statistically significant (*p* = 0.33) [1].

A meta-analysis by Esagian et al. demonstrated a significantly shorter LOS for RAMIE compared to OTE. The mean LOS was 17.10 ± 9.39 days for RAMIE versus 30.68 ± 23.88 days for OTE, with a weighted mean difference (WMD) of −9.22 days (95% CI: −14.39 to −4.06; *p* < 0.001) [24]. In the RAMIE trial, the median ICU stay remained consistent at one day across both RAMIE (range 0–15) and MIE (range 0–14) groups (*p* = 0.990). Similarly, the median postoperative hospital stay was nine days for both groups (RAMIE: range 6–49; MIE: range 6–82; *p* = 0.311). Additionally, the readmission rate to the ICU was identical between the groups (1.7% each; *p* = 0.815) [23].

The meta-analysis by Perry et al., encompassing 26 studies, further supported the shorter LOS associated with RAMIE. The mean LOS was 18.57 days for RAMIE versus 33.11 days for cMIE, yielding a statistically significant mean difference of −3.03 days (95% CI: −4.51 to −1.54; *p* < 0.0001; I^2^ = 96%; *p* < 0.00001) [25]. Finally, the meta-analysis by Zhou et al. specifically focused on McKeown esophagectomy, highlighting a shorter postoperative hospital stay for RAMIE compared to cMIE (MD = 1.05 days, 95% CI: 0.05–2.05; *p* = 0.04) [26].

#### 3.1.2. Functional Recovery

Postoperative functional recovery is a critical outcome measure following esophagectomy. In the ROBOT trial, functional recovery was defined as the successful removal of thoracic tubes, absence of intravenous fluid resuscitation requirements, tolerance for solid oral intake, independent mobilization, and adequate pain control with oral analgesics. At postoperative day 14, significantly more patients in the RAMIE group achieved functional recovery compared to those in the OTE group (38/54, 70% vs. 28/55, 51%; RR 1.48, 95% CI: 1.03–2.13; *p* = 0.04) [1]. These findings underscore the potential advantages of robotic-assisted techniques in promoting faster recovery and reducing the burden of postoperative care.

#### 3.1.3. Postoperative Pain Management

In the ROBOT trial, postoperative pain was assessed using a visual analogue scale (VAS) ranging from 1 to 10, with measurements taken preoperatively and daily during the first 14 days post-surgery. Patients in the RAMIE group reported significantly lower mean pain scores compared to the OTE group (1.86 vs. 2.62; *p* < 0.001). Furthermore, short-term quality-of-life (QoL) outcomes were superior in the RAMIE group at both discharge and six weeks post-discharge. The mean difference in QoL scores between the groups was 13.4 (95% CI: 2.0–24.7; *p* = 0.02) at discharge and 11.1 (95% CI: 1.0–21.1; *p* = 0.03) at six weeks post-discharge. Physical functioning also demonstrated significant improvement in the RAMIE group compared to the OTE group, with mean differences of 13.5 (95% CI: 1.2–25.7; *p* = 0.03) at discharge and 10.7 (95% CI: 0.04–21.4; *p* = 0.049) at six weeks. These findings highlight the role of robotic-assisted techniques in reducing postoperative discomfort and enhancing the overall recovery experience for patients [1].

### 3.2. Complications

#### 3.2.1. Pulmonary Complications

Robotic-assisted surgery offers superior anatomical visualization and enhanced dexterity, enabling precise preservation of parasympathetic lung innervation through sparing of vagal branches. This has been linked to a reduction in pulmonary complications [28].

In the ROBOT trial, pulmonary complications were significantly lower in the RAMIE group (17/54 patients, 32%) compared to the OTE group (32/55 patients, 58%), with a relative risk (RR) of 0.54 (95% CI: 0.34–0.85; *p* = 0.005) [1]. Similarly, the meta-analysis by Esagian et al. revealed a significantly lower overall pulmonary complication rate in the RAMIE group (14.29%, 49/343) versus the OTE group (25.32%, 174/687), with an odds ratio (OR) of 0.38 (95% CI: 0.26–0.56; *p* < 0.001) [24].

However, in the RAMIE trial, the incidence of pulmonary complications was comparable between RAMIE (13.8%) and MIE (14.7%) (RR 0.94, 95% CI: 0.57–1.56; *p* = 0.812). No significant differences were observed in the rates of pneumonia (9.9% in RAMIE vs. 11.9% in MIE; *p* = 0.560) or respiratory failure (4.4% in RAMIE vs. 5.1% in MIE; *p* = 0.767) [23].

The meta-analysis by Perry et al. analyzed 10,154 patients (RAMIE: 3185; cMIE: 6969) and found no statistically significant difference in pulmonary complication rates between RAMIE (20.13%) and cMIE (22.20%) (RR 0.89, 95% CI: 0.77–1.02; *p* = 0.10; I^2^ = 18%; *p* = 0.20) [25]. In contrast, the meta-analysis by Zhou et al. reported a considerably lower risk of pneumonia following RAMIE compared to cMIE (OR 0.72, 95% CI: 0.52–1.00; *p* = 0.05) [26]. Similarly, a meta-analysis comparing Ivor Lewis RAMIE with cMIE found a lower weighted pooled proportion of postoperative pneumonia in RAMIE (8%, 95% CI: 6–9) than in cMIE (10%, 95% CI: 7–13). Patients undergoing RAMIE demonstrated a significantly reduced risk of pneumonia (OR 0.46, 95% CI: 0.35–0.61; *p* < 0.0001) [27].

#### 3.2.2. Cardiac Complications

Cardiac complications, particularly new-onset atrial fibrillation (AF), are associated with significant postoperative morbidity and may serve as surrogate markers for underlying complications. The reduced incidence of cardiac complications in robotic-assisted minimally invasive esophagectomy (RAMIE) may be attributed to decreased intravascular depletion due to less blood loss, reduced oxidative stress from superior lung ventilation in the prone position, and fewer infectious complications compared to open transthoracic esophagectomy (OTE) [29].

In the ROBOT trial, atrial fibrillation occurred in 22% of patients in the RAMIE group (17/45) compared to 47% in the OTE group (26/55), yielding a relative risk (RR) of 0.47 (95% CI: 0.27–0.83; *p* = 0.006) [1]. Similarly, the meta-analysis by Esagian et al., which included data from five studies [30,31], reported a significantly lower rate of atrial fibrillation in the RAMIE group (6.79%, 29/427) compared to the OTE group (8.46%, 54/638), with an odds ratio (OR) of 0.53 (95% CI: 0.29–0.98; *p* = 0.04) [24]. Conversely, the RAMIE trial found comparable rates of cardiac complications in the RAMIE and conventional minimally invasive esophagectomy (cMIE) groups (two cases vs. one case, *p* = 0.8) [23].

**Figure 1:**
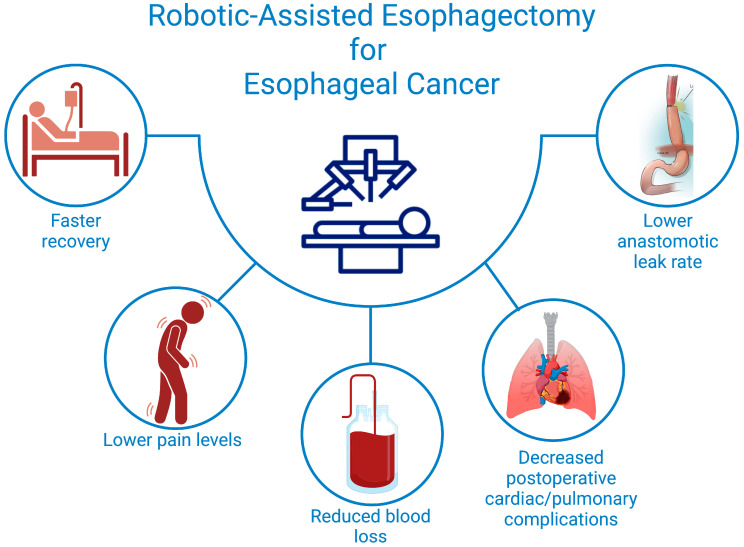
Postoperative Advantages of Robotic-Assisted Esophagectomy for Better Patient Outcomes.

The meta-analysis by Perry et al. further supported this finding, with cardiac complication rates of 14.02% (365/2604) in the RAMIE group and 15.74% (823/5228) in the cMIE group. No statistically significant difference was observed between the two groups (RR 1.01, 95% CI: 0.86–1.19; *p* = 0.88; I^2^ = 3%, *p* = 0.42) [25] (Figure 1).

#### 3.2.3. Anastomotic Leak

The Esophageal Complications Consensus Group (ECCG) defines an anastomotic leak as a “full-thickness gastrointestinal defect involving the esophagus, anastomosis, staple line, or conduit irrespective of presentation or method of identification.” The ECCG further classifies leaks into three types based on the intervention required for management [20]. The technical challenges in crafting a gastric conduit during McKeown esophagectomy and creating an intrathoracic anastomosis in Ivor Lewis esophagectomy are exacerbated in conventional minimally invasive esophagectomy (cMIE) due to the limited maneuverability of laparoscopic instruments. Robotic-assisted minimally invasive esophagectomy (RAMIE) addresses these challenges with enhanced ergonomics, tremor filtration, and the precise control offered by EndoWrist instruments [27].

In a retrospective analysis by de Groot et al., 26% of 152 patients undergoing RAMIE with intrathoracic anastomosis experienced anastomotic leaks. Of these 40 patients, 28% had management failure, including seven cases of esophagobronchial fistula, three cases of anastomotic disconnection, and one death due to septic bleeding [32]. Similarly, Khaitan et al., using data from the Society of Thoracic Surgeons General Thoracic Surgery Database, found that RAMIE was independently associated with higher rates of anastomotic leak compared to OTE (adjusted odds ratio [aOR] 1.53, 95% CI: 1.14–2.04) [33]. In a propensity-matched analysis of 1320 RAMIE cases versus cMIE, the rate of anastomotic leaks requiring surgery was higher in the RAMIE group (aOR 1.39, 95% CI: 1.01–1.92; *p* = 0.045). Excessive handling of the gastric conduit by robotic instruments, coupled with lower case volumes and the learning curve of surgeons, was suggested as a potential reason for the higher leak rates [33].

In the ROBOT trial, type III anastomotic leaks occurred in 22% of patients in the RAMIE arm and 20% in the OTE arm (*p* = 0.57) [1]. A meta-analysis by Esagian et al. found no statistically significant difference in leak rates between RAMIE (6.82%; 46/674) and OTE (6.06%; 79/1303), with an odds ratio of 0.93 (95% CI: 0.60–1.44; *p* = 0.76) [24]. Similarly, the RAMIE trial reported comparable rates of anastomotic leakage between RAMIE (12.2%) and cMIE (11.3%) (RR 1.08; 95% CI: 0.61–1.90; *p* = 0.801), with only one patient in each group requiring surgical intervention for type III leakage [23].

The meta-analysis by Perry et al. reported anastomotic leak rates of 12.47% (391/3136) in the RAMIE group and 11.43% (785/6866) in the cMIE group, favoring cMIE (RR 1.23; 95% CI: 1.09–1.38; *p* = 0.0005; I^2^ = 0%, *p* = 0.64) [25]. A meta-analysis comparing Ivor Lewis cMIE and RAMIE found comparable leak rates, with 7% (95% CI: 6–9%) in the cMIE group and 7% (95% CI: 5–11%) in the RAMIE group. The odds of developing an anastomotic leak were similar between the two groups (OR 0.85; 95% CI: 0.65–1.10; *p* = 0.22) [27] (Table 1).

#### 3.2.4. Gastric Conduit Necrosis

The Esophageal Complications Consensus Group (ECCG) classifies gastric conduit necrosis into three types based on the extent of necrosis and the requirement for surgical management and diversion [20]. In the ROBOT trial, type III gastric conduit necrosis was observed in one patient in the RAMIE arm and two patients in the OTE arm (*p* = 1.00) [1].

#### 3.2.5. Thoracic Duct

Chylothorax, a significant complication following esophagectomy, is associated with substantial morbidity, often requiring prolonged management and impacting patient recovery. The identification of the thoracic duct is critical in minimizing the risk of chylothorax, and recent advances in near-infrared fluorescence (NIRF) imaging have improved the precision of this process. The use of NIRF for thoracic duct identification has been well established in non-robotic esophageal surgeries [34,35], and its integration into robotic-assisted procedures holds promise for enhancing surgical outcomes.

A study by Vecchiato et al. in 2020 proposed an innovative approach for detecting the thoracic duct during robotic or laparoscopic surgery. This technique involved intranodal injection of indocyanine green (ICG) into the inguinal nodes under ultrasound guidance, followed by robotic or laparoscopic detection of the thoracic duct in 21 patients. The procedure was successful in identifying the thoracic duct in all cases, with one intraoperative injury to the duct that was promptly clipped. Importantly, no postoperative chylothorax or adverse reactions at the injection site were observed, underscoring the safety and effectiveness of this technique [35].

In addition, Jardinet et al. demonstrated the feasibility of this approach with intrainguinal node ICG injection, offering easy identification of the thoracic duct during surgery. This video publication further reinforces the potential of NIRF-guided identification to prevent thoracic duct injury and subsequent complications such as chylothorax [36].

These findings suggest that NIRF imaging, combined with the intranodal injection of ICG, is a promising method for safely identifying the thoracic duct in robotic esophagectomy. By minimizing the risk of injury and improving the precision of lymphatic structure identification, this approach may reduce postoperative complications and improve patient outcomes. Further studies are necessary to refine this technique and assess its broader applicability in clinical practice.

#### 3.2.6. Chyle Leak

The ECCG defines chyle leaks by classifying them into three types and grades of severity based on daily output [20]. Dezube et al., in a retrospective analysis, compared 70 RAMIE procedures with 277 cMIE procedures, reporting a significantly higher incidence of chyle leaks in the RAMIE group compared to the cMIE group (12.8% vs. 3.6%, *p* = 0.006) [37]. Notably, McKeown RAMIE was associated with a higher incidence of chyle leaks (33%) than Ivor Lewis RAMIE (4%).

In the ROBOT trial, chyle leaks were documented in 17 patients in the RAMIE arm and 12 patients in the OTE arm, with no statistically significant difference (*p* = 0.69) [1]. Similarly, the meta-analysis by Esagian et al. showed no significant difference in the incidence of chylothorax between the RAMIE group (5.39%; 29/538) and the OTE group (3.01%; 33/1095), with an odds ratio of 1.31 (95% CI: 0.75–2.29; *p* = 0.35) [24].

The RAMIE trial also reported comparable rates of chyle leak between RAMIE (five cases) and cMIE (two cases) (*p* = 0.449) [23]. Perry et al., in their meta-analysis, found no statistically significant difference in chyle leak rates between the two groups (RR 1.07; 95% CI: 0.72–1.60; *p* = 0.74; I^2^ = 13%, *p* = 0.30). The leak rates were 2.82% (69/2443) in the RAMIE group and 3.84% (197/5135) in the cMIE group [25]. Similarly, Zhou et al. observed no significant difference in pooled data analysis between RAMIE and cMIE approaches (OR: 1.33; 95% CI: 0.57–3.07; *p* = 0.51) [26].

#### 3.2.7. Recurrent Laryngeal Nerve (RLN) Injury

During esophagectomy, the recurrent laryngeal nerve (RLN) is vulnerable to injury due to thermal damage, stretching, compression, or vascular compromise, potentially leading to vocal cord palsy. Such injuries can significantly increase the risk of pulmonary complications, ICU readmissions, and prolonged hospital stays [33]. The ECCG defines vocal cord injury as “vocal cord dysfunction post-resection, confirmed and assessed by direct examination”, with severity graded by laterality and intervention needed, categorized into three types [21]. Robotics has been hypothesized to reduce RLN injuries due to improved visualization of the nerve and adjacent vascular structures.

A retrospective analysis by Scholtemeijer et al. on McKeown esophagectomies reported an RLN injury incidence of 14% among 266 patients undergoing robotic-assisted minimally invasive esophagectomy (RAMIE) [38].

In the ROBOT trial, type 1 vocal cord injury was observed in five patients in the RAMIE arm and six patients in the OTE arm, with no statistically significant difference (*p* = 0.78) [23]. Similarly, the meta-analysis by Esagian et al., which included seven studies, found no significant difference in RLN palsy rates between RAMIE (13.99%; 67/479) and OTE (10.41%; 84/807) groups (OR: 1.31, 95% CI: 0.90–1.90; *p* = 0.16) [24].

In the RAMIE trial, RAMIE was associated with a higher rate of vocal cord paralysis compared to cMIE (32.6% vs. 27.1%), although the difference was not statistically significant (RR 1.20; 95% CI: 0.87–1.66; *p* = 0.258) [23]. The largest meta-analysis, conducted by Perry et al., included over 18,000 patients and reported no significant difference in RLN injury rates between RAMIE (8.94%; 237/2652) and cMIE (7.63%; 423/5541). The relative risk was 0.96 (95% CI: 0.82–1.13; *p* = 0.62; I^2^ = 7%; *p* = 0.36) [25].

#### 3.2.8. Para-Conduit Diaphragmatic Herniations

Para-conduit diaphragmatic herniation is a relatively rare complication following open esophagectomy. However, with the increasing adoption of minimally invasive techniques, the incidence of this complication has significantly risen. This trend is likely attributable to the reduction in adhesions between the gastric conduit and the diaphragmatic hiatus inherent to minimally invasive approaches. In a retrospective analysis by De Silva et al. [30], the incidence of para-conduit diaphragmatic herniation was found to be significantly higher with minimally invasive techniques (laparoscopic and robotic approaches) compared to open esophagectomy (70.8% vs. 35.5%; *p* < 0.001). To reduce the risk of herniation, it has been suggested that surgeons place two or three interrupted sutures between the gastric conduit and the right hemidiaphragm after completing the anastomosis. This maneuver may provide additional stability and prevent the herniation of abdominal contents into the thoracic cavity [30].

### 3.3. Postoperative Mortality

In the ROBOT trial, two patients on the RAMIE arm and one patient on the OTE arm died in the immediate postoperative period, and 30-day and 90-day mortality rates were 2% and 9% in the RAMIE arm vs. 0% and 2% in the OTE arm [24].

In the RAMIE trial, for 30-day mortality, one patient in MIE died from acute cerebral infarction on POD 12. For 90-day mortality, one patient in RAMIE died from severe pneumonia on POD 42 [23]. 

In the meta-analysis by Perry et al., the 30-day mortality rate for each procedure was 1.63% (44/2707) in the RAMIE group and 1.87% (117/6244) in the cMIE group. There was no statistically significant difference between the two groups (RR 1.03, *p* = 0.88 [95% CI 0.73, 1.44], I^2^ = 0%, *p* = 0.53). The rate of 90-day mortality was 3.55% (106/2987) in the RAMIE group and 4.84% (336/6946) in the cMIE group, showing no statistically significant difference between the two groups (RR 0.95, *p* = 0.66 [95% CI 0.77, 1.18], I^2^ = 0%, *p* = 0.93) [25]. In the meta-analysis by Zhou et al., the in-hospital mortality and 90-day mortality had no statistically distinguished difference in a merged data analysis (OR = 0.54, 95 CI [0.14, 2.02] *p* = 0.36) and (OR = 0.69, 95 CI [0.26, 1.83] *p* = 0.46), respectively [26].

## 4. Fluorescence-Guided Technologies in Robotic Esophageal Cancer Surgery

Fluorescence-guided surgery has revolutionized many surgical specialties by enabling more precise and safer procedures. In esophageal cancer surgery, a particularly complex domain, this technology has become an essential tool for enhancing surgical outcomes. This outlines the current applications of fluorescence in identifying lymphatic structures, performing sentinel node biopsy, visualizing the thoracic duct, and conducting angiography during robotic-assisted esophagectomy.

### 4.1. Angiography

Anastomotic leakage following esophagectomy is a significant complication, occurring in 6-41% of patients and associated with considerable morbidity and mortality [39]. Fluorescence angiography has proven effective in reducing the incidence of such complications by providing real-time vascular visualization. In a study of 30 patients, Sarkaria et al. demonstrated the utility of fluorescence in identifying the termination of the vascular arcade and small transverse vessels under fluorescence, which aided in confirming the vascular supply during the mobilization of the greater curvature and omentum [40].

In a larger cohort of 75 patients, Egberts et al. utilized fluorescence angiography to analyze gastric conduit perfusion during robotic surgery [31]. While the majority of patients benefited from this technique, Hodari et al. reported anastomotic leakage in three patients, even with real-time perfusion assessment [41]. Similar studies conducted by Pötscher et al. [42] and DeLong et al. [43] corroborate the efficacy of fluorescence in detecting perfusion issues, although challenges remain in predicting leaks with absolute certainty.

Slooter et al., in their study of 81 patients undergoing Ivory Lewis and McKeown esophagectomies with robotic assistance, found that the time interval between indocyanine green (ICG) injection and conduit tip reinforcement was a significant predictor of outcomes, with a cut-off value of 98 s [44]. In open surgery, Ishikawa et al. proposed a quantitative analysis using three parameters—ingress index at both the tip and 5 cm of the conduit, and ingress time—as key indicators for predicting anastomotic leaks following esophagectomy [45]. These findings suggest that a more quantitative approach to fluorescence angiography could enhance the prediction and prevention of postoperative complications.

### 4.2. Near-Infrared Fluorescent-Guided Lymphadenectomy and Sentinel Node Biopsy

Lymphadenectomy plays a crucial role in achieving optimal oncological outcomes in esophageal cancer surgery. The use of image-guided lymphadenectomy has been well established in non-robotic surgery [46,47], and recent advancements in robotic-assisted esophagectomy have integrated near-infrared fluorescence (NIRF) imaging for more precise identification and resection of lymphatic structures. This technique enhances the surgeon’s ability to visualize lymph nodes, especially in areas that are difficult to access or identify through conventional methods.

In a study by Hosogi et al., 15 patients undergoing robotic esophagectomy were assessed for NIRF-guided lymphadenectomy. The study found that 80% of patients had NIRF-stained lymph nodes in the right recurrent laryngeal nerve area, and 73% had stained lymph nodes in the left recurrent laryngeal nerve area, highlighting the ability of NIRF imaging to facilitate accurate lymph node mapping during robotic surgery [48]. Furthermore, the prospective ESOMAP feasibility trial, which evaluated robotic-assisted minimally invasive Ivory Lewis esophagectomy, demonstrated the feasibility of intraoperative NIRF-guided lymph node mapping and resection for pathological examination. In a cohort of 20 patients, 5 had no ICG uptake during a standard D2 lymphadenectomy, but notably, the NIRF-guided procedures were significantly shorter compared to non-NIRF procedures, suggesting potential advantages in terms of operative efficiency.

The study of NIRF-stained lymph nodes in gastroesophageal junction cancer showed no increase in the number of harvested lymph nodes compared to a historical control group. Additionally, there were no significant differences in operative time, blood loss, or other postoperative complications between the NIRF and non-NIRF groups [49]. These findings suggest that while NIRF-guided lymphadenectomy may improve the precision of lymph node identification, it may not necessarily result in a higher number of harvested nodes or improved clinical outcomes in all cases.

In a study by Shiomi et al., 54 patients without preoperative chemotherapy were divided into groups based on NIRF-guided resection, with ICG-positive or -negative lymph nodes and metastasis-positive or -negative nodes. This study revealed that preoperative chemotherapy affected the sensitivity of NIRF in predicting metastatic lymph nodes, indicating that the effectiveness of NIRF imaging may vary depending on the patient’s treatment history [50].

A hybrid approach combining technetium-99m and ICG for sentinel node biopsy (SNB) was also investigated in high-risk pT1c patients. In a cohort of 10 patients, the hybrid tracer successfully identified sentinel nodes in all cases. In one patient, the dissection was found to be incomplete, and in four patients, additional fluorescent lymph nodes were harvested, with micrometastases identified in two cases [51]. Similarly, Overwater et al. applied a hybrid tracer in minimally invasive surgery, including robotic surgery, for pT1b esophageal cancer patients. They found successful sentinel node identification in all five patients, with two cases revealing additional peritumoral fluorescent-only sentinel nodes, though no postoperative pathological metastases were found [52].

These studies underscore the promising role of NIRF in esophageal cancer surgery, particularly for sentinel node biopsy and lymphadenectomy. However, challenges remain, such as the impact of preoperative chemotherapy on the sensitivity of NIRF in detecting metastatic lymph nodes. Further research and refinement of these techniques will be essential to determine their full clinical potential and to standardize their use in esophageal cancer treatment protocols.

## 5. Postoperative Outcomes of Newer Robotic Approaches

The evolution of robotic surgical techniques has introduced innovative approaches aimed at minimizing surgical trauma while maintaining or improving patient outcomes. One such method, the robotic transhiatal approach (Th-RAMIE), has been designed to eliminate the need for thoracotomy or single-lung ventilation, thereby reducing the associated morbidity. In a prospective trial conducted by Williams et al., 97 patients undergoing Th-RAMIE were compared to 212 patients treated with open transhiatal esophagectomy (THE) for patient-related outcomes. The study demonstrated comparable outcomes between the two groups; however, opioid use at discharge was significantly lower in the Th-RAMIE group (71% vs. 82%; *p* = 0.03) [53]. These findings suggest that Th-RAMIE offers an advantage in postoperative pain management, potentially enhancing recovery while preserving oncological outcomes.

### 5.1. Robotic-Assisted Cervical Esophagectomy (RACE)

Another promising advancement is the single-port trans-cervical approach, also known as robotic-assisted cervical esophagectomy (RACE). This novel technique is gaining traction as an alternative to the conventional thoracic approach, with the potential to markedly reduce pulmonary complications by completely avoiding thoracic incisions [54]. The articulated robotic arms provide enhanced maneuverability and precision, reducing interference with critical structures such as the recurrent laryngeal nerve. The three-dimensional, magnified field of view inherent to robotic platforms allows for a more accurate assessment of anatomical relationships, which may lead to improved patient outcomes and quality of life.

Fujita et al. reported on a case series of ten patients who underwent robot-assisted trans-cervical esophagectomy using a bilateral cervical approach. The short-term postoperative outcomes revealed the following complication rates: arrhythmia in 10.0% of patients, vocal cord paralysis in 10.0%, anastomotic leakage in 20.0%, and no cases of postoperative pneumonia [38]. While the study highlights the feasibility of this approach, it underscores the need for larger studies to further evaluate its safety profile and long-term outcomes.

### 5.2. Future Implications

The ongoing ROBOT-2 and REVATE trials are expected to provide valuable insights into the benefits of robotic approaches in esophageal cancer surgery. The ROBOT-2 trial, which compares robot-assisted minimally invasive thoraco-laparoscopic esophagectomy with conventional minimally invasive esophagectomy for resectable esophageal adenocarcinoma, aims to further elucidate the potential advantages of robotic surgery in terms of postoperative recovery and long-term oncological outcomes [38]. Similarly, the REVATE trial, which contrasts robotic-assisted esophagectomy with video-assisted thoracoscopic esophagectomy (VATS), will help clarify the relative merits of robotic systems in minimizing complications, enhancing surgical precision, and improving functional recovery [55].

These trials play a crucial role in expanding the evidence base regarding the impact of robotic techniques on postoperative outcomes, particularly with regard to complication rates, recovery timelines, and overall patient quality of life. As the data from these studies accumulate, they may refine current clinical practice and offer stronger evidence for the adoption of robotic approaches in esophageal surgery.

## 6. Conclusions

In conclusion, robotic-assisted approaches to esophageal cancer surgery have shown significant improvements in surgical precision, postoperative recovery, and complication management. The integration of robotic techniques, such as RAMIE, Th-RAMIE, and RACE, along with fluorescence-guided technologies, has enhanced lymphadenectomy, sentinel node biopsy, and thoracic duct identification, contributing to better oncological outcomes and reduced morbidity. Studies consistently demonstrate that robotic surgery offers advantages such as lower postoperative pain, faster functional recovery, and fewer complications like anastomotic leakage and chylothorax, compared to traditional open approaches. As ongoing trials continue to evaluate long-term outcomes, the evidence strongly supports robotic surgery as a superior modality for esophagectomy, offering both clinical benefits and the potential for improved patient quality of life.

## Figures and Tables

**Table 1:** Comparative Analysis of Anastomotic Leak Rates Across Surgical Approaches and Study Types.

Author(s)	Study Type	Sample Size	Procedure/Comparison	Anastomotic Leak Rate (%)	*p*-Value	Key Findings
De Groot et al., 2023 [32]	Retrospective Analysis	152 patients	RAMIE with Intrathoracic Anastomosis	26	Not reported	High leak rate, particularly with esophagobronchial fistula.
Khaitan et al., 2023 [33]	Database Analysis	Society Database: 1320 RAMIE vs. 3524 cMIE vs. 5763 OTE	RAMIE vs. CMIE vs. OTE	Higher in RAMIE (Adjusted OR: 1.53)	*p* = 0.045	Higher leak rate in RAMIE linked to learning curve and lower volumes.
Van der Sluis et al., 2019 [1]	Randomized Controlled Trial (RCT)	54 RAMIE vs. 55 OTE	RAMIE vs. OTE	22 (RAMIE), 20 (OTE)	*p* = 0.57	No significant difference in leak rates between RAMIE and OTE.
Esagian et al., 2022 [24]	Meta-Analysis	674 RAMIE vs. 1303 OTE	RAMIE vs. OTE	6.82 (RAMIE), 6.06 (OTE)	*p* = 0.76	No statistical significance in leak rates between groups.
Yang et al., 2022 [23]	Randomized Controlled Trial (RCT)	RAMIE Trial: 50 patients in each group	RAMIE vs. MIE	12.2 (RAMIE), 11.3 (MIE)	*p* = 0.801	Similar leak rates in RAMIE and MIE with low surgical interventions.
Perry et al., 2024 [25]	Meta-Analysis	3136 RAMIE vs. 6866 cMIE	RAMIE vs. cMIE	12.47 (RAMIE), 11.43 (cMIE)	*p* = 0.005	Higher leak rate in RAMIE group, favoring cMIE.
Zhou et al., 2022 [26]	Meta-Analysis	Pooled Data: 7 studies, sample sizes not specified	Ivor Lewis RAMIE vs. cMIE	Comparable (7% each group)	*p* = 0.22	Leak rates were comparable, no significant difference found.

RAMIE: robotic-assisted minimally invasive esophagectomy; OTE: open transthoracic esophagectomy; cMIE: conventional minimally invasive esophagectomy; OR: odds ratio; RCT: randomized controlled trial.

## Data Availability

The datasets generated and/or analyzed during this study are not publicly available but may be obtained from the corresponding author upon reasonable request.
